# Estimated glomerular filtration rate and risk of all‐cause mortality

**DOI:** 10.1111/1753-0407.13513

**Published:** 2023-12-04

**Authors:** Tomoyuki Kawada

**Affiliations:** ^1^ Department of Hygiene and Public Health Nippon Medical School Tokyo Japan

Dear Editor,

Liu et al.^1^ conducted a prospective study to examine the associations between several estimated glomerular filtration rates (eGFRs) and all‐cause mortality with special reference to diabetes status. The mean age of the subjects was about 60 years, and eGFR measure was classified into three groups: normal, modestly declined, and chronic kidney disease (CKD). The adjusted hazard ratio (HR) of CKD defined by eGFRs with cystatin C and a combination with cystatin C and creatinine for all‐cause mortality significantly increased among patients with diabetes. In contrast, the adjusted HR of modestly declined eGFR and CKD with use of any estimated equations for all‐cause mortality significantly increased. I have some concerns about their study.

First, Hussain et al.^2^ investigated age‐specific associations of eGFR with adverse outcomes. By setting age‐specific eGFR reference as a control, the adjusted HR (95% CI) of modest reduction in eGFRs for composite adverse outcome (all‐cause mortality, any cardiovascular event, and kidney failure) in subjects aged 18–39, 40–49, and 50–65 were 1.42 (1.35–1.49), 1.13 (1.10–1.16), and 1.08 (1.07–1.09), respectively. Although the absolute risk in the incidence of composite adverse outcome in subjects with mild reduction of eGFR increased by aging, special attention should be paid to the younger generation for composite adverse outcome. They used serum creatinine for estimating eGFR, and I suppose that additional analyses by using cystatin C and a combination with cystatin C and creatinine should be conducted, with special reference to diabetes status. Anyway, the aging effect may be important for the adverse health outcomes by decreased eGFR.

Second, the Chronic Kidney Disease Prognosis Consortium^3^ had reported about a meta‐analysis that HRs of eGFR and urinary albumin–creatinine ratio (UACR) for all‐cause mortality were higher in women than men. Losito et al.^4^ investigated the association of reduced kidney function, diabetes, and arterial hypertension with mortality in patients with cardiovascular disease. All‐cause mortality was significantly associated with aging, male sex, and reduced eGFR only in patients with chronic ischemic heart disease, and diabetes or arterial hypertension did not contribute to the association. I recommend further studies to evaluate the mortality risk by reduced eGFR with special reference to sex difference because there are sex differences in the lifestyle factors, several clinical outcomes, and predictive equation of eGFR. Regarding the interaction of diabetes on the association, other comorbidities might also contribute to the association between reduced eGFR and subsequent all‐cause mortality. Anyway, the magnitude of each comorbidity on the association should be examined.

## FUNDING INFORMATION

There is no financial support for this paper.

## DISCLOSURE

The author declares that he has no competing interests. No ethical statement is needed for this paper.
